# Supplementary material for: Interactions between Nanoparticles and Intestine
Source: Int J Mol Sci. 2022 Apr 14;23(8):4339. doi: 10.3390/ijms23084339 (PMC9024817; doi:10.3390/ijms23084339)
Supplement: Supplementary file 1 [file ijms-23-04339-s001.zip › ijms-1645968-supplementary.pdf]

Review

Interactions between nanoparticles and intestine

Manuela Vitulo [1†], Elisa Gnodi [1†], Raffaella Meneveri [1] and Donatella Barisani [1,*]

[1] School of Medicine and Surgery, University of Milano-Bicocca, 20900 Monza, Italy; m.vitulo1@campus.unimib.it (M.V.); e.gnodi@campus.unimib.it (E.G.); raffaella.meneveri@unimib.it (R.M.).
* Correspondence: donatella.barisani@unimib.it; (D.B.) Tel.: +39 0264488304
† These authors contributed equally to this work

Table S1. List of FDA and EMA approved nanomedicines

Drug name	Company	Material Used	Approval Date	Administration route
		Polymer-based Nanoparticles		
**Adagen®**	Enzon Pharmaceuticals Inc.	PEGylated- Adenosine deaminase (ADA)	FDA (1990)	Injectable
**Copaxone®/FOGA**	Teva Pharmaceutical Industries Ltd.	Mannitol-glatiramer acetate	FDA (1996), EMA (2016)	Injectable
**Neulasta®**	Amgen, Inc.	PEGylated GCSF protein	FDA (2002)	Injectable
**PegIntron®**	Merk & Co. Inc.	PEGylated- alpha interferon (INF) molecule	EMA (2000), FDA (2001)	Injectable
**Pegasys®**	Genentech USA, Inc.	PEGylated-recombinant human IFN alpha-2a protein	FDA, EMA (2002)	Injectable
**Eligard®**	Tolmar Pharmaceuticals Inc.	Leuprolide acetate and polymer (PLGH (poly (DL-Lactide-coglycolide))	FDA (2002)	Injectable
**Somavert®**	Pfizer Pharmaceuticals	PEGylated HGH receptor antagonist	EMA (2002), FDA (2003)	Injectable
**Macugen®**	Pfizer Pharmaceuticals	PEGylated anti-VEGF aptamer	FDA (2004)	Injectable
**Cimzia®**	UCB	PEGylated- IgG Fab' fragment	FDA (2008), EMA (2009)	Injectable
**Plegridy®**	Biogene)	PEGylated- recombinant IFN-	FDA (2014)	Injectable
**Zilretta®**	Flexion Therapeutics	PLGA- Triamcinolone acetonide	FDA (2017)	Injectable
**Oncaspar®**	Enzon Pharmaceuticals Inc.	Polymer-protein conjugate (PEGylated L-asparaginase)	FDA (1994), EMA (2016)	Injectable/intravenous
**Mircera®**	Vifor	PEGylated- ESA (erythropoiesis-stimulating agent)	EMA (2007), FDA (2018)	Injectable/intravenous
**Diprivan®**	Fresenius Kabi	Soybean oil based- Propofol	FDA (1989), EMA (2001)	Intravenous
**Genexol-PM®**	Lupin Ltd.	Micelles loaded- Paclitaxel	FDA (2007)	Intravenous
**Krystexxa®**	Savient Pharmaceuticals	Polymer-protein conjugate (PEGylated porcine-like	FDA (2010)	Intravenous

		uricase)		
**Adynovate®**	Baxalta US Inc.	PEGylated- coagulation factor VIII	FDA (2015)	Intravenous
**Rebinyn®**	NovoNordisk	Glyco-PEGylated- DNA-derived coagulation FIX	FDA (2017)	Intravenous
**Apealea®**	Oasmia Pharmaceutical AB	Micelles loaded- Paclitaxel	EMA (2018)	Intravenous
**Renagel®**	Sanofi	Colloidal Silicon dioxide	FDA (2000)	oral
**Renagel®/Renvela®**	Genzyme	Poly(allylamine hydrochloride)	EMA (2007)	oral
**Restasis®**	Allergan	Carbon copolymer type A-Cyclosporine	FDA (2003)	topical
**Estrasorb™**	Novavax, Inc.	Micellar Estradiol	FDA (2003)	topical
		Lipid-based Nanoparticles		
**Abelcet®**	Defiante Farmaceutica	Liposomal Amphotericin B lipid complex	FDA (1995)	Intravenous
**DaunoXome®**	Galen Ltd.	Liposomal Daunorubicin	FDA, EMA (1996)	Intravenous
**Caelyx®**	Janssen Pharmaceuticals	Pegylated- liposomal Doxorubicin	EMA (1996)	Intravenous
**Doxil®**	Johnson & Johnson	Liposomal Doxorubicin	FDA (1995), EMA (1996)	Intravenous
**AmBisome®**	NeXstar Pharmaceuticals	Liposomal Amphotericin B	EMA (1990), FDA (1997)	Intravenous
**Myocet®**	Teva Pharmaceutical Industries Ltd.	Liposomal Doxorubicin hydrochloride	EMA (2000)	Intravenous
**Zevalin®**	Bayer Pharma	Liposomal 90Y- Ibritumomab tiuxetan	EMA (2004)	Intravenous
**DepoDur®**	SkyePharma	Liposomal morphine sulphate	FDA (2004), EMA (2006)	Intravenous
**Mepact®**	Takeda France SAS	Liposomal Mifamurtide	EMA (2009)	Intravenous
**Marqibo®**	Talon Therapeutics	Liposomal Vincristine	FDA (2012)	Intravenous
**Lipodox®**	Sun Pharma Global FZE	PEGylated- Liposomal Doxorubicin hydrochloride	FDA (2013)	Intravenous
**Onivyde®**	Merrimack Pharmaceuticals	Liposomal Irinotecan	FDA (2015)	Intravenous
**Lipusu®**	/	Liposomal Paclitaxel	FDA (2016)	Intravenous
**Vyxeos®**	Jazz Pharmaceutics	Liposomal Daunorubicin and Cytarabine	FDA (2017), EMA (2018)	Intravenous
**Onpattro®**	Alnylam	PEGylated Liposomal Patisiran	FDA & EMA (2018)	Intravenous
**DepoCyt®**	Pacira Pharmaceuticals	Liposomal Cytarabine	EMA (2002), FDA (2007)	Injectable
**Inflexal®**	Crucell Berna Biotech	Viral Liposomal- inactivated influenza virus vaccine	EMA (1997)	Injectable
**Pfizer-BioNTech Vaccine**	Pfizer Pharmaceuticals	mRNA vaccine	FDA (2020)	Injectable
**Moderna COVID-19 Vaccine**	ModernaTX Inc.	mRNA vaccine	FDA (2020)	Injectable
**Curosurf®**	Chiesi	Liposome-proteins SP-B and SP-C	FDA (1999)	Intra-tracheal

		Inorganic Nanoparticles		
**Dexferrum®**	American Regent	Iron dextran	FDA (1996)	Intravenous
**Ferrlecit®**	Sanofi-Aventis	Sodium ferric gluconate	FDA (1999), EMA (2013)	Intravenous
**Venofer®**	Luitpold Pharm	Iron sucrose	FDA (2000)	Intravenous
**Feraheme™**	AMAG Pharmaceuticals	Ferumoxytol	FDA (2009)	Intravenous
**Ferinject®**	Vifor	Iron carboxymaltose colloid	FDA, EMA (2013)	Injectable/intravenous
**Infed®**	Actavis Pharma	iron dextran	FDA (1992)	Injectable/intravenous
**Hensify®**	Nanobiotix	Hafnium oxide nanoparticles	EMA (2019)	Injectable/intravenous
		Dendrimer-based Nanoparticles		
**VivaGel® BV**	Starpharma	Astodrimer sodium	FDA (2015)	Topical
		Protein-based Nanoparticles		
**Ontak®**	Eisai	Protein combining diphtheria toxin and IL-2	FDA (1999)	Intravenous
**Abraxane®**	Celgene Pharmaceutical Co. Ltd.	Albumin-bound Paclitaxel	FDA (2005, 2012, 2013), EMA (2008)	Intravenous

Data were taken from www.clinicaltrials.gov accessed on 1 April 2022.
